# *PLOS ONE* 2018 Reviewer and Editorial Board Thank You

**DOI:** 10.1371/journal.pone.0213287

**Published:** 2019-02-27

PLOS and the *PLOS ONE* team want to sincerely thank all of our Editorial Board Members, Guest Editors, and Reviewers for the journal in 2018. Your contributions of time and expertise support your research community, advance scientific progress, and continue to make *PLOS ONE* a leader in its field. This past year, *PLOS ONE* received the assistance of more than 8,000 Editorial Board members, 900 Guest Editors, and 53,000 Reviewers, who handled more than 36,000 manuscripts that resulted in 17,000 publications (Fig 1).

**Fig 1:**
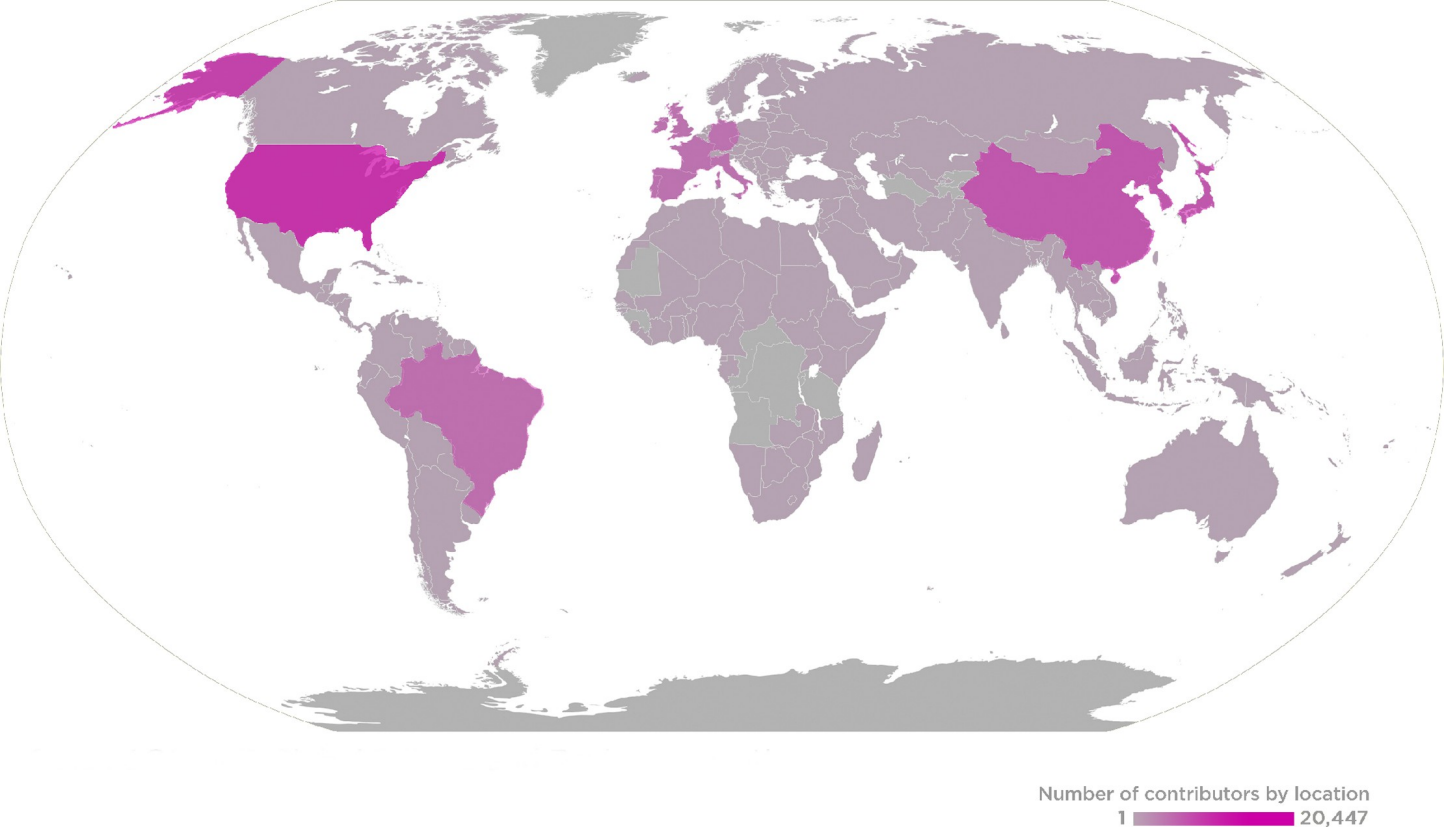
2018 *PLOS ONE* Global Editor and Reviewer Locations.

We’re deeply grateful to all of our volunteers whose dedicated efforts support *PLOS ONE* and Open Science. Thank you all for your work!

## References

[1] (2018) *PLOS ONE* 2017 Reviewer and Editorial Board Thank You. PLoS ONE 13(3): e0194158 10.1371/journal.pone.0194158 29543840

[2] (2017) *PLOS ONE* 2016 Reviewer and Editorial Board Thank You. PLoS ONE 12(3): e0174259 10.1371/journal.pone.0174259

[3] (2016) *PLOS ONE* 2015 Reviewer Thank You. PLoS ONE 11(2): e0150341 10.1371/journal.pone.0150341

[4] (2015) *PLOS ONE* 2014 Reviewer Thank You. PLoS ONE 10(2): e0121093 10.1371/journal.pone.0121093

